# FDG PET metabolic signatures distinguishing prodromal DLB and prodromal AD

**DOI:** 10.1016/j.nicl.2021.102754

**Published:** 2021-07-04

**Authors:** Kejal Kantarci, Bradley F. Boeve, Scott A. Przybelski, Timothy G. Lesnick, Qin Chen, Julie Fields, Christopher G. Schwarz, Matthew L. Senjem, Jeffrey L. Gunte, Clifford R. Jack, Paul Min, Manoj Jain, Toji Migayawa, Rodolfo Savica, Jonathan Graff-Radford, Hugo Botha, David T. Jones, David S. Knopman, Neill Graff-Radford, Tanis J. Ferman, Ronald C. Petersen, Val J. Lowe

**Affiliations:** a Department of Radiology, Mayo Clinic, Rochester, MN, USA; b Department of Neurology, Mayo Clinic, Rochester, MN, USA; c Department of Health Sciences Research, Mayo Clinic, Rochester, MN, USA; d Department of Psychiatry and Psychology, Mayo Clinic, Rochester, MN, USA; e Department of Information Technology, Mayo Clinic, Rochester, MN, USA; f Department of Radiology, Mayo Clinic, Jacksonville, FL, USA; g Department of Neurology, Mayo Clinic, Jacksonville, FL, USA; h Department of Psychiatry and Psychology, Mayo Clinic, Jacksonville, FL, USA

**Keywords:** FDG PET, Mild cognitive impairment, Demenia with Lewy bodies, Alzheimer's disease, Cingulate island sign

## Abstract

**Background and Purpose:**

Patients with dementia with Lewy bodies (DLB) are characterized by hypometabolism in the parieto–occipital cortex and the cingulate island sign (CIS) on ^18^F-fluorodeoxyglucose (FDG) PET. Whether this pattern of hypometabolism is present as early as the prodromal stage of DLB is unknown. We investigated the pattern of hypometabolism in patients with mild cognitive impairment (MCI) who progressed to probable DLB compared to MCI patients who progressed to Alzheimer’s disease (AD) dementia and clinically unimpaired (CU) controls.

**Methods:**

Patients with MCI from the Mayo Clinic Alzheimer’s Disease Research Center who underwent FDG PET at baseline and progressed to either probable DLB (MCI-DLB; n = 17) or AD dementia (MCI-AD; n = 41) during follow-up, and a comparison cohort of CU controls (n = 100) were included.

**Results:**

Patients with MCI-DLB had hypometabolism in the parieto-occipital cortex extending into temporal lobes, substantia nigra and thalamus. When compared to MCI-AD, medial temporal and posterior cingulate metabolism were preserved in patients with MCI-DLB, accompanied by greater hypometabolism in the substantia nigra in MCI-DLB compared to MCI-AD. In distinguishing MCI-DLB from MCI-AD at the maximum value of Youden’s index, CIS ratio was highly specific (90%) but not sensitive (59%), but a higher medial temporal to substantia nigra ratio was both sensitive (94%) and specific (83%).

**Conclusion:**

FDG PET is a potential biomarker for the prodromal stage of DLB. A higher medial temporal metabolism and CIS ratio, and lower substantia nigra metabolism have additive value in distinguishing prodromal DLB and AD.

## Highlights

• FDG PET distinguish prodromal DLB from prodromal Alzheimer’s disease.
• The topography of hypometabolism in prodromal DLB is consistent with DLB pattern.
• Medial temporal /substantia nigra FDG ratio provided excellent discrimination.

## 1 Introduction

Prodromal dementia with Lewy bodies (DLB) is the pre-dementia phase of DLB, and a proportion of the prodromal DLB patients can be characterized by mild cognitive impairment (MCI) with core features of DLB (McKeith et al., 2020). Imaging biomarkers that show abnormalities at the prodromal stage, and that distinguish between patients with MCI who will progress to probable DLB from those who progress to Alzheimer’s disease (AD) dementia are needed for the evaluation of effective treatments early in the disease course, and to help avoid the iatrogenic and potentially deadly effects of other agents.

^18^F-fluorodeoxyglucose (FDG) PET findings in patients with DLB are characterized by a parieto-occipital pattern of hypometabolism and a relatively preserved posterior cingulate metabolism known as the cingulate island sign (CIS) (Lim et al., 2009). CIS have been particularly effective in distinguishing patients with probable DLB from AD dementia in clinical cohorts as well as pathologically confirmed AD and Lewy body disease cohorts with 83% sensitivity and 93% specificity (Lim et al., 2009, Graff-Radford et al., 2014, Graff-Radford et al., 2020). Whether these hypometabolic patterns are present during the prodromal stage in DLB and whether FDG PET can accurately distinguish DLB from AD during the prodromal stage is currently unknown (Massa et al., 2019, Boeve, 2012). Moreover, it is possible that other regional patterns of hypometabolism present during the prodromal stage may be more accurate in distinguishing prodromal DLB and AD patients compared to the regional patterns commonly observed during the dementia stage.

In the current study, we investigated the pattern of hypometabolism on FDG PET in patients with MCI who progressed to probable DLB (MCI-DLB) compared to a clinically unimpaired (CU) cohort of controls. We further compared MCI-DLB to MCI patients who progressed to AD dementia (MCI-AD) with the objective of identifying a metabolic signature that distinguishes prodromal DLB from prodromal AD.

## 2 Methods

### 2.1 Participants

The subjects of this study were identified from the Mayo Clinic Alzheimer’s Disease Research Center (ADRC). We studied patients diagnosed with MCI who underwent FDG PET imaging along with an MRI examination at baseline from 2006 until 2018 (n = 148) and progressed to either probable DLB (n = 17), or AD dementia (n = 41) during approximately annual follow-ups through 2019. Because this was a study on the FDG PET differences between prodromal DLB and prodromal AD, MCI patients who converted to neurodegenerative diseases other than AD or DLB (n = 11; e.g. familial or sporadic FTLD), who did not return for clinical follow-up (n = 27), or who did not progress to dementia (n = 56) were excluded. Diagnosis of MCI was made according to the criteria by Petersen et al. (Petersen et al., 2013, Petersen, 2004). Diagnosis of AD was made according to the NIA-AA criteria (McKhann et al., 2011). Probable DLB patients fulfilled the 4th Consortium Criteria during the longitudinal clinical evaluation (McKeith et al., 2017). Biomarkers or imaging findings were not utilized during clinical assessment and diagnosis. Clinically unimpaired participants from the Mayo Clinic Study of Aging (MCSA), which is a population-based cohort from Olmstead County, MN (Roberts et al., 2008) were included as the control group. The control group was frequency matched on age and sex to the MCI participants of the current study (n = 100).

Assessments for the clinical features of DLB have been detailed in previous reports from the ADRC cohorts (Roberts et al., 2008, Kantarci et al., 2012). Briefly, presence of parkinsonism was determined from neurologic examination, and motor severity was assessed using the Unified Parkinson’s Disease Rating Scale part III. Visual hallucinations were characterized by being fully formed, were not restricted to a single episode and were not related to another medical issue, treatment or advanced dementia. Fluctuations were considered to be present if the patients scored 3 to 4 on the 4-item Mayo Fluctuations Scale (Ferman et al., 2004). Probable rapid eye movement sleep behavior disorder (RBD) met the International Classification of Sleep Disorders-II diagnostic criteria B for probable RBD (AASM, 2005).

### 2.2 MRI and FDG PET imaging

MRI examinations were performed at 3 Tesla (GE Healthcare). A 3-D high resolution magnetization prepared rapid gradient echo acquisition with approximately 1 mm cubic resolution was obtained for anatomical segmentation and labeling. PET/computed tomography scanners (GE Healthcare) operating in 3-dimensional mode were used to acquire PET images. Patients were injected with an average of 296 MBq (range, 266– 326 MBq) FDG. Following a 30 min FDG uptake period, four 3.75 min frames were obtained.

### 2.3 FDG PET image analysis

FDG PET image analysis was performed using an automated image processing pipeline, which included rigid registration of the PET image volumes to each subject’s own 3-dimensional T1-weighted MRI using SPM12. MR images were segmented using Unified Segmentation in SPM12 with population-optimized priors and settings from the Mayo Clinic Adult Lifespan Template. FDG uptake in each voxel was referenced to the median value of the pons uptake for the standardized uptake value ratio (SUVr) in each voxel. Regional cortical uptake of FDG was determined using the DISTAL atlas (Ewert et al., 2018) for the substantia nigra region, and the Mayo Clinic Adult Lifespan Template Atlas (Schwarz et al., 2017) for all other regions. CIS ratio was calculated by dividing the posterior cingulate uptake to precuneus and cuneus FDG uptake value. A voxel based analysis (VBM) was conducted in SPM12 comparing FDG SUVr in the MCI-DLB group to both the CU and the MCI-AD and the MCI-AD group to the CU group. Maps of these comparisons were displayed at the p < 0.001 level. Correction for multiple comparisons was applied using family wise error (FWE) correction and if no differences were observed, uncorrected maps were displayed.

### 2.4 Statistical analysis

Characteristics of the subjects were described using means and standard deviations for continuous variables, and counts and percentages for categorical variables. Differences in characteristics of the three groups were evaluated using one-way analysis of variance (ANOVA) or Fisher's exact test. Additional pairwise-comparisons were done using contrast statements for continuous variables or pairwise Fisher’s exact tests for categorical variables. FDG SUVr in regions that distinguish MCI-DLB and MCI-AD on voxel-based analysis were included in univariate and then multivariate logistic regression models of two and three variables. Receiver operating characteristic (ROC) curves were generated for the models, and the area under the ROC (AUROC) value was reported for each model as a measure of each model’s ability to distinguish MCI-DLB and MCI-AD. Sensitivity and specificity values were calculated for predicted probability cut-offs across the ROC curves, and the maximum Youden’s index, which maximizes the distance from the ROC curve to the identity line, was calculated as a summary statistic.

## 3 Results

### 3.1 Characteristics of the cohort

Characteristics of the cohort classified by the clinical group are listed in Table 1. The MCI-DLB and MCI-AD groups did not differ in age at imaging or the Clinical Dementia Rating Sum of Boxes scores at the time of imaging, but the frequency of women was higher in the MCI-AD (39%) than the MCI-DLB, which included only male patients (p = 0.003). The CU group on average was similar to the two MCI groups on age and sex by design. The MCI-AD group on average had lower Mini Mental State Examination scores than the MCI-DLB group (p < 0.001). MCI-AD group was followed on average for 2 years before progression to AD, shorter than the MCI-DLB who were followed for 3.2 years before progression to DLB, but this difference was not statistically significant (p = 0.08). Whereas a majority of the MCI-DLB patients had parkinsonism (82%) and RBD (88%), or both (71%) at the time of imaging, frequency of visual hallucinations (35%) and fluctuations (41%) was less common.

**Table 1:** Characteristics of participants.

	CUn = 100	MCI-DLBn = 17	MCI-ADn = 41	P-value^a^
Age, yrs	69.7 (9.4)	68.6 (5.6)	70.5 (8.7)	0.75
Males, no. (%)	72 (72%)	17 (100%)	25 (61%)	0.004
APOE4, no. (%)	32 (32%)	6 (35%)	28 (70%)	<0.001
Education, yrs	15.3 (2.7)	16.2 (2.7)	16.1 (3.1)	0.20
CDR Sum of boxes	0.0 (0.1)	1.8 (1.0)	1.7 (1.1)	<0.001
MMSE	28.7 (0.9)	27.7 (2.7)	26.0 (2.7)	<0.001
Follow-up, yrs		3.2 (1.9)	2.0 (1.3)	0.08
UPDRS Part III		13.8 (12.1)	1.0 (2.3)	<0.001
DRS Total		135.4 (6.6)	131.1 (7.6)	0.049
Visual Hallucinations, no. (%)		6 (35%)	0 (0%)	<0.001
Fluctuations, no. (%)		7 (41%)	0 (0%)	<0.001
Parkinsonism, no. (%)		14 (82%)	3 (7%)	<0.001
RBD, no. (%)		15 (88%)	5 (12%)	<0.001

Mean (SD) listed for the continuous variables and count (%) for the categorical variables. UPDRS Total and DRS Total values are not reported in the clinically unimpaired participants from the Mayo Clinic Study of Aging.

CU: clinically unimpaired. CDR: Clinical Dementia Rating Scale, MMSE: Mini-Mental State Examination, UPDRS: Unified Parkinson’s Disease Rating Scale (UPDRS), DRS: Mattis Dementia Rating Scale, pRBD: probable REM Sleep Behavior Disorder.

a P-values represent an ANOVA test for continuous variables and Fisher’s exact test for categorical variables.

### 3.2 FDG PET findings in the MCI-DLB and MCI-AD groups

**Fig. 1:**
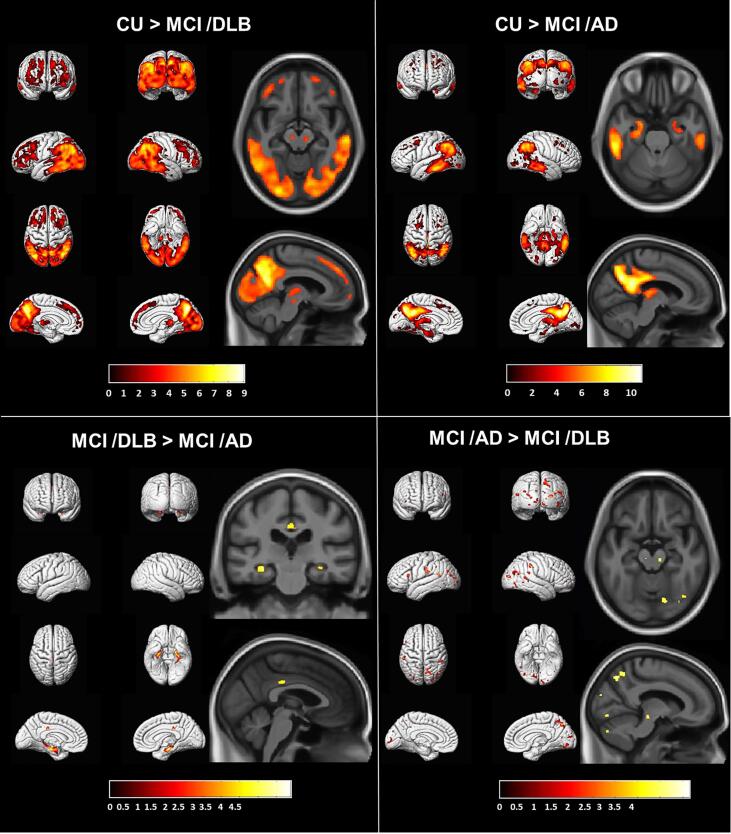
Voxel-based analysis comparing FDG PET SUVr in the MCI-DLB, MCI-AD and clinically unimpaired (CU) controls. Maps of these comparisons were displayed at the p < 0.001 level with the t-values displayed in the color bar. The voxel-based regional differences in FDG SUVr in MCI groups compared to CU were corrected for multiple comparisons using family-wise error correction. Because there were no differences identified between the MCI-DLB and MCI-AD groups after correction for multiple comparisons, we display the uncorrected results.

The voxel-based regional differences in FDG SUVr in MCI groups compared to CU (p < 0.001; corrected for multiple comparisons using FWE) are displayed in Fig. 1. The MCI-DLB group had lower FDG uptake in the posterior temporal, parietal and occipital lobes, and to a lesser extent in the frontal lobes compared to the CU group, with relative sparing of the anterior and medial temporal lobes and the primary sensory and motor cortices. Furthermore, thalamus and substantia nigra showed hypometabolism in the MCI-DLB group compared to the CU. Medial temporal hypometabolism along with temporal and parietal hypometabolism with relative sparing of the frontal and occipital lobes were observed in the MCI-AD group compared to CU. Subcortically, thalamus was also involved with hypometabolism in MCI-AD compared to CU.

Fig. 1 shows the differences in cortical FDG SUVr among the MCI-DLB and MCI-AD groups. Because there were no differences identified between the two groups after correction for multiple comparisons, we display the uncorrected results (p < 0.001). Patients with MCI-AD had lower FDG uptake in the medial temporal lobe and in the posterior-mid cingulate cortex bilaterally compared to MCI-DLB. On the other hand, patients with MCI-DLB had lower FDG uptake in the substantia nigra and the parietal and occipital regions bilaterally compared to MCI-AD.

**Fig. 2:**
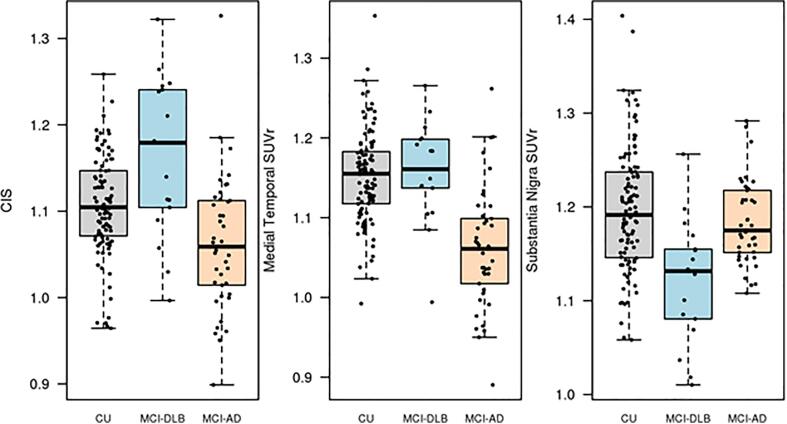
Box Plots demonstrating cingulate island sign ratio (CISr), medial temporal SUVr, and substantia nigra SUVr from the atlas-based analysis.

Data from atlas-based analysis with boxplots showing these differences in CIS ratio, medial temporal (hippocampus, parahippocampal/entorhinal cortex, and amygdala) and substantia nigra SUVrs are displayed in Fig. 2**.**

Univariate logistic regression revealed that each of the three measurements had a greater likelihood of predicting MCI-DLB compared to MCI-AD, and multivariable modeling showed that measurement pairs were predictive of MCI-DLB compared to MCI-AD when adjusting for each other. The AUROCs improved when two and three variables were considered together. Whereas all variables contributed when included in two variable models, only the medial temporal and substantia nigra SUVrs significantly contributed in the three variable model and the CIS ratio did not reach statistical significance (Table 2).

**Table 2:** Logistic regression modeling of imaging predictor variables.

	OR (95% CI)^a^	AUROC	P-value
*Single biomarker predictors*			
**CIS ratio**	4.06 (1.74, 9.45)	0.793	0.001
**Medial Temporal SUVr**	6.37 (2.50, 20.98)	0.844	<0.001
**Substantia Nigra SUVr**	0.09 (0.02, 0.41)	0.791	0.002
*Two biomarker predictors*			
**CIS ratio + Medial Temporal SUVr**			
CIS ratio	2.67 (1.12, 6.37)	0.884	0.026
Medial Temporal SUVr	4.33 (1.44, 13.00)		0.009
**CIS ratio + Substantia Nigra SUVr**			
CIS ratio	4.14 (1.60, 10.67)	0.875	0.003
Substantia Nigra SUVr	0.10 (0.02, 0.52)		0.006
**Medial Temporal + Substantia Nigra SUVrs**			
Medial Temporal SUVr	6.60 (1.95, 22.31)	0.902	0.002
Substantia Nigra SUVr	0.10 (0.02, 0.53)		0.007
*All three biomarker predictors*			
**CIS ratio + Medial Temporal + Substantia Nigra SUVrs**		0.921	
CIS ratio	2.56 (0.84, 7.78)		0.097
Medial Temporal	3.86 (1.10, 13.49)		0.035
Substantia Nigra	0.13 (0.02, 0.70)		0.018

a In the logistic regression analysis, the odds ratios are based on 0.10 unit change. MCI-AD is the reference group. When OR is greater than 1 the values are higher in the MCI-DLB and conversely when the OR is<1 then the values are higher in MCI-AD individuals.

**Fig. 3:**
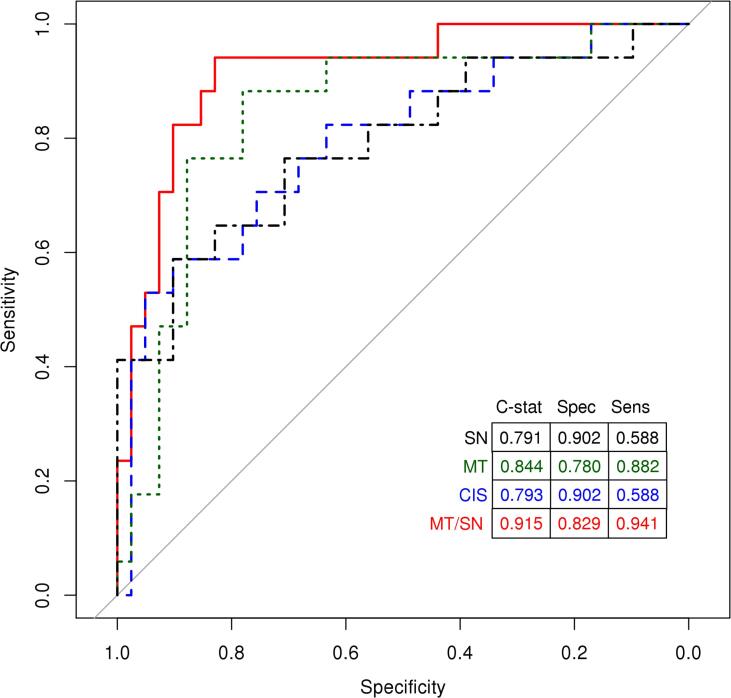
Receiver operating characteristic analysis. Sensitivity and specificity was calculated using Youden’s index, which maximizes the distance to the identity line.

Given the higher medial temporal SUVr and lower substantia nigra SUVr in MCI-DLB compared to MCI-AD, in the univariate and multivariable models, we combined the two measures and investigated the diagnostic accuracy of the ratio of medial temporal SUVr substantia nigra SUVr. Fig. 3 shows the ROC curves for distinguishing MCI-DLB and MCI-AD using CIS ratio, medial temporal and substantia nigra SUVrs as well as the medial temporal/substantia nigra SUVr ratio. In distinguishing MCI-DLB from MCI-AD, at the maximum value of Youden’s Index, CIS ratio was high, although sensitivity was low (sensitivity of 59% and specificity of 90%), while the medial temporal to substantia nigra SUVr ratio had high sensitivity and specificity (sensitivity of 94% and specificity of 83%) with the highest accuracy (AUROC of 91.5%).

## 4 Discussion

This study demonstrated that MCI patients who progress to probable DLB (MCI-DLB) have wide-spread hypometabolism on FDG PET compared to the CU controls. The topographic pattern of hypometabolism observed in MCI-DLB, involving posterior cortical regions including the occipital lobes as well as thalamus was consistent with the pattern observed in DLB (Lim et al., 2009, Graff-Radford et al., 2014, Albin et al., 1996, Imamura et al., 1997, Ishii et al., 1998, Koeppe et al., 2005, Mosconi et al., 2008, Pillai et al., 2019). Furthermore, the quantitative CIS ratio was higher in MCI-DLB than MCI-AD, indicating that FDG PET findings that distinguish probable DLB and AD dementia are present as early as the prodromal stage in MCI patients. Besides these typical patterns observed in MCI-DLB and MCI-AD, we found that the substantia nigra FDG uptake was lower, and medial temporal lobe FDG uptake was higher in MCI-DLB compared to MCI-AD and these two variables combined as the medial temporal /substantia nigra ratio provided excellent discrimination of MCI-DLB from MCI-AD.

FDG PET has been accurate in distinguishing clinically diagnosed probable DLB and AD dementia patients, as well as distinguishing pathologically confirmed LBD from AD (Lim et al., 2009, Graff-Radford et al., 2014, Albin et al., 1996, Imamura et al., 1997, Ishii et al., 1998, Koeppe et al., 2005, Mosconi et al., 2008, Pillai et al., 2019). Based on these observations, occipital hypometabolism together with CIS was classified as a supportive biomarker of DLB in the 4th Consortium Criteria (McKeith et al., 2017). In particular, the relative preservation of FDG uptake in the posterior portion of the cingulate gyrus (CIS) has accurately identified cases with LBD who did not have additional AD pathology (Lim et al., 2009, Graff-Radford et al., 2014, Kantarci et al., 2012, Pillai et al., 2019). The magnitude of the CIS quantified with the CIS ratio is associated with the neurofibrillary tangle (NFT)-tau stage at autopsy, such that higher Braak NFT stage at autopsy correlates with a lower antemortem CIS ratio in patients with DLB (Graff-Radford et al., 2020). In the current study, CIS ratio in MCI-DLB was higher than MCI-AD. In addition, voxel-based analysis revealed higher FDG uptake in the posterior-mid cingulate region in MCI-DLB compared to MCI-AD, This finding, according to our knowledge, has not been demonstrated when comparing patients with probable DLB and AD dementia using voxel-based analysis (Graff-Radford et al., 2014, Kantarci et al., 2012).

AD pathology mixed with LBD is common in patients with probable DLB and a subset of patients with probable DLB fulfill the pathologic criteria for the diagnosis of LBD and AD, and also exhibit antemortem AD biomarker positivity (Kantarci et al., 2020). It is possible that MCI-DLB patients have lower levels of NFT tau pathology than patients with probable DLB. In that case, CIS may be more apparent on FDG PET scans and CIS ratio would have a higher accuracy in distinguishing between DLB and AD in early stages of the disease, including during the prodromal stage. The clinical progression of patients with mixed pathology is faster (Graff-Radford et al., 2020); and as such, it is possible that this group may transition through MCI faster and may be underrepresented in the current cohort. It is also possible that pathological heterogeneity increases with disease progression from MCI-DLB to probable DLB. A CIS ratio that accurately distinguishes MCI-DLB and MCI-AD as demonstrated in voxel-based analysis suggests a lower frequency of mixed pathology in MCI-DLB than probable DLB patients. This makes CIS ratio a potential biomarker to distinguish MCI patients who will progress to probable DLB versus probable AD.

Preservation of medial temporal lobe volume is a supportive biomarker for the diagnosis of probable DLB (McKeith et al., 2017), and predicts progression to probable DLB versus probable AD in patients with MCI (Kantarci et al., 2016). Similarly, preservation of medial temporal metabolism has been proposed as a potential biomarker for distinguishing probable DLB from other dementias such as AD dementia and frontotemporal lobar degeneration (Pillai et al., 2019, Oppedal et al., 2019). When hippocampal atrophy is present in DLB, it predicts greater NFT tau pathology and a faster cognitive decline (Graff-Radford et al., 2016, Kantarci et al., 2012). We observed preservation of medial temporal lobe metabolism in MCI-DLB compared to MCI-AD, which is consistent with findings in probable DLB, suggesting that medial temporal metabolic signature may have value in distinguishing AD dementia and DLB during the prodromal stage.

One of the main pathways connecting medial temporal lobe and posterior cingulate cortex is the cingulum bundle. Posterior cingulate hypometabolism is associated with hippocampal atrophy through disruption of the cingulum bundle in AD dementia (Villain et al., 2008). Hence, posterior cingulate hypometabolism is thought to have a remote association with neurodegeneration in the hippocampus through the connecting pathways. Contrary to AD dementia, hippocampal and cingulum bundle structures are preserved in probable DLB (Kantarci et al., 2010). Preservation of the posterior cingulate and the medial temporal lobe metabolism in MCI-DLB compared to MCI-AD is in alignment with the structural connectivity of these regions and our data indicates that both of these regional findings additively improve the diagnostic performance of FDG PET during the prodromal stage.

Neurodegeneration of the substantia nigra and associated decrease in nigrostriatal dopaminergic input to the basal ganglia is a characteristic feature of Lewy body disease, and is observed on dopamine transporter SPECT imaging in patients with prodromal DLB (Thomas et al., 2019). Current results provide evidence of lower substantia nigra metabolism in patients with MCI who later progressed to probable DLB. This pattern of reduction in substantia nigra metabolism has also been reported in Parkinson’s disease (Ruppert et al., 2020). Our findings suggest that hypometabolism in the substantia nigra is an early biomarker of Lewy body disease. Substantia nigra hypometabolism could not be compared between MCI-DLB patients with and without parkinsonism because only three MCI-DLB patients did not have parkinsonism. Further research is needed to understand substantia nigra hypometabolism in the MCI stage of DLB in patients without parkinsonism, and to better characterize the temporal characteristics of the loss of dopaminergic activity in prodromal stages of DLB

Multivariate logistic regression analysis demonstrated that multiple regional measurements from the FDG PET scans improved the diagnostic accuracy in distinguishing MCI-DLB and MCI-AD. In the three-variable model (substantia nigra SUVR, medial temporal lobe SUVR, and CIS ratio), only lower substantia nigra and higher medial temporal lobe metabolism contributed to the model and improved the diagnostic accuracy in distinguishing MCI-DLB from MCI-AD. Therefore, we combined the two measurements as the substantia nigra /medial temporal lobe uptake ratio, which had the highest accuracy (AUROC of 93%) with sensitivity of 94% and specificity of 83% at the maximum value of Youden’s Index. Although the ratio of the uptake in these two structures may be quite accurate in distinguishing the two clinical groups cross-sectionally, future research on test–retest reproducibility and longitudinal reliability of these measurements are needed.

The clinically classified MCI-DLB and MCI-AD patients all progressed to probable DLB or AD dementia at follow-up by design. In our effort to promote the discovery of new biomarkers for prodromal DLB, we limited the study to this specific subset of ADRC-based MCI who progressed to DLB or AD and did not include the broad range of all MCI patients in a time-to-event analysis (Petersen et al., 2013, Kantarci et al., 2010). We acknowledge several limitations of our study. One limitation was that by design we had to include MCI patients who progressed to dementia during the follow-up period, therefore the MCI patients were more severely impaired than expected. Furthermore, we did not utilize biomarkers for the classification of the MCI groups and relied on clinical diagnosis due to lack of availability of biomarkers in all participants. Another limitation was that the cohort of MCI-DLB patients were men. Although men are generally represented more frequently in the Lewy body disease cohorts, one possibility is that the disease has a slower pace in men than women, therefore men tend to be captured at the MCI stage more often than women. Higher prevalence of MCI in men compared to women was previously observed in a population-based cohort (Roberts et al., 2012). Because the study cohort was relatively small, particularly for MCI-DLB group, the FDG PET signatures of prodromal DLB identified in this study will need to be corroborated in independent cohorts (Oppedal et al., 2019), and tested in epidemiologic and clinic-based MCI cohorts to determine their utility in predicting progression to AD dementia or probable DLB.

## 5 Conclusions

Current data demonstrates that FDG PET abnormalities are present as early as the prodromal phase of DLB and regional FDG PET signatures can accurately distinguish prodromal DLB and prodromal AD patients. Because of the profound hypometabolism observed in MCI-DLB patients, it is imperative to determine the longitudinal metabolic change in cohorts that are at-risk for DLB, such as patients with idiopathic RBD and MCI patients with the core clinical features of DLB, to determine the temporal occurrence of regional hypometabolism and clinical progression.

## 6 Data availability statement

Anonymized data can be provided upon review of the request. Data sharing agreement with the requesting investigator and their institution is required.

## Funding

This study was funded by the NIH (U01 NS100620, P50 AG16574, U01 AG06786, R01 AG11378, and C06 RR018898), Foundation Dr. Corinne Schulerand, the Mangurian Foundation for Lewy Body Research, The Elsie and Marvin Dekelboum Family Foundation, and the Robert H. and Clarice Smith and Abigail Van Buren Alzheimer’s Disease Research Program. The funding sources had no role in study design, collection, analysis, interpretation, or decision to submit this paper. The corresponding author had full access to all the data in the study and had final responsibility for the decision to submit for publication.

## Declaration of Competing Interest

The authors declare that they have no known competing financial interests or personal relationships that could have appeared to influence the work reported in this paper.

## References

[b0005] McKeith I.G., Ferman T.J., Thomas A.J., Blanc F., Boeve B.F., Fujishiro H., Kantarci K., Muscio C., O'Brien J.T., Postuma R.B., Aarsland D., Ballard C., Bonanni L., Donaghy P., Emre M., Galvin J.E., Galasko D., Goldman J.G., Gomperts S.N., Honig L.S., Ikeda M., Leverenz J.B., Lewis S.J.G., Marder K.S., Masellis M., Salmon D.P., Taylor J.P., Tsuang D.W., Walker Z., Tiraboschi P. (2020). Prodromal DLBDSG: Research criteria for the diagnosis of prodromal dementia with Lewy bodies. Neurology.

[b0010] Lim S.M., Katsifis A., Villemagne V.L., Best R., Jones G., Saling M., Bradshaw J., Merory J., Woodward M., Hopwood M., Rowe C.C. (2009). The 18F-FDG PET cingulate island sign and comparison to 123I-beta-CIT SPECT for diagnosis of dementia with Lewy bodies. J. Nucl. Med. Off. Publ. Soc. Nucl. Med..

[b0015] Graff-Radford J., Murray M.E., Lowe V.J., Boeve B.F., Ferman T.J., Przybelski S.A., Lesnick T.G., Senjem M.L., Gunter J.L., Smith G.E., Knopman D.S., Jack C.R., Dickson D.W., Petersen R.C., Kantarci K. (2014). Dementia with Lewy bodies: Basis of cingulate island sign. Neurology.

[b0020] Graff-Radford J., Lesnick T.G., Savica R., Chen Q., Ferman T.J., Przybelski S.A., Jones D.T., Senjem M.L., Gunter J.L., Kremers W.K., Jack C.R., Lowe V.J., Petersen R.C., Knopman D.S., Boeve B.F., Murray M.E., Dickson D.W., Kantarci K. (2020). (18)F-fluorodeoxyglucose positron emission tomography in dementia with Lewy bodies. Brain Commun..

[b0025] Massa F., Arnaldi D., De Cesari F., Girtler N., Brugnolo A., Grazzini M., Bauckneht M., Meli R., Morbelli S., Pardini M., Sambuceti G., De Carli F., Tiraboschi P., Nobili F. (2019). Neuroimaging findings and clinical trajectories of Lewy body disease in patients with MCI. Neurobiol. Aging.

[b0030] Boeve B.F. (2012). Mild cognitive impairment associated with underlying Alzheimer's disease versus Lewy body disease. Parkinsonism Related Disord..

[b0035] Petersen R.C., Aisen P., Boeve B.F., Geda Y.E., Ivnik R.J., Knopman D.S., Mielke M., Pankratz V.S., Roberts R., Rocca W.A., Weigand S., Weiner M., Wiste H., Jack C.R. (2013). Criteria for mild cognitive impairment due to alzheimer's disease in the community. Ann. Neurol..

[b0040] Petersen R.C. (2004). Mild cognitive impairment as a diagnostic entity. J. Intern. Med..

[b0045] McKhann G.M., Knopman D.S., Chertkow H., Hyman B.T., Jack C.R., Kawas C.H., Klunk W.E., Koroshetz W.J., Manly J.J., Mayeux R., Mohs R.C., Morris J.C., Rossor M.N., Scheltens P., Carrillo M.C., Thies B., Weintraub S., Phelps C.H. (2011). The diagnosis of dementia due to Alzheimer's disease: recommendations from the National Institute on Aging-Alzheimer's Association workgroups on diagnostic guidelines for Alzheimer's disease. Alzheimer's Dementia J. Alzheimer's Assoc..

[b0050] McKeith I.G., Boeve B.F., Dickson D.W., Halliday G., Taylor J.P., Weintraub D., Aarsland D., Galvin J., Attems J., Ballard C.G., Bayston A., Beach T.G., Blanc F., Bohnen N., Bonanni L., Bras J., Brundin P., Burn D., Chen-Plotkin A., Duda J.E., El-Agnaf O., Feldman H., Ferman T.J., Ffytche D., Fujishiro H., Galasko D., Goldman J.G., Gomperts S.N., Graff-Radford N.R., Honig L.S., Iranzo A., Kantarci K., Kaufer D., Kukull W., Lee V.M.Y., Leverenz J.B., Lewis S., Lippa C., Lunde A., Masellis M., Masliah E., McLean P., Mollenhauer B., Montine T.J., Moreno E., Mori E., Murray M., O'Brien J.T., Orimo S., Postuma R.B., Ramaswamy S., Ross O.A., Salmon D.P., Singleton A., Taylor A., Thomas A., Tiraboschi P., Toledo J.B., Trojanowski J.Q., Tsuang D., Walker Z., Yamada M., Kosaka K. (2017). Diagnosis and management of dementia with Lewy bodies: Fourth consensus report of the DLB Consortium. Neurology.

[b0055] Roberts R.O., Geda Y.E., Knopman D.S., Cha R.H., Pankratz V.S., Boeve B.F., Ivnik R.J., Tangalos E.G., Petersen R.C., Rocca W.A. (2008). The Mayo clinic study of aging: Design and sampling, participation, baseline measures and sample characteristics. Neuroepidemiology.

[b0060] Kantarci K., Lowe V.J., Boeve B.F., Weigand S.D., Senjem M.L., Przybelski S.A., Dickson D.W., Parisi J.E., Knopman D.S., Smith G.E., Ferman T.J., Petersen R.C., Jack C.R. (2012). Multimodality imaging characteristics of dementia with Lewy bodies. Neurobiol. Aging.

[b0065] Ferman T.J., Smith G.E., Boeve B.F., Ivnik R.J., Petersen R.C., Knopman D., Graff-Radford N., Parisi J., Dickson D.W. (2004). DLB fluctuations: specific features that reliably differentiate DLB from AD and normal aging. Neurology.

[b0070] AASM: International Classification of Sleep Disorders—2: Diagnostic and Coding Manual. American Academy of Sleep Medicine, Chicago. 2005.

[b0075] Ewert S., Plettig P., Li N., Chakravarty M.M., Collins D.L., Herrington T.M., Kuhn A.A., Horn A. (2018). Toward defining deep brain stimulation targets in MNI space: A subcortical atlas based on multimodal MRI, histology and structural connectivity. Neuroimage.

[b0080] Schwarz C.G., Gunter J.L.W.C., Vemuri P., Senjem M.L., Wiste H.J., Petersen R.C., Knopman D.S. (2017). CR J: The Mayo Clinic adult lifespan template: Better quantification across the lifespan. Alzheimer's Dementia J. Alzheimer's Assoc..

[b0085] Albin R.L., Minoshima S., D'Amato C.J., Frey K.A., Kuhl D.A., Sima A.A. (1996). Fluoro-deoxyglucose positron emission tomography in diffuse Lewy body disease. Neurology.

[b0090] Imamura T., Ishii K., Sasaki M., Kitagaki H., Yamaji S., Hirono N., Shimomura T., Hashimoto M., Tanimukai S., Kazui H., Mori E. (1997). Regional cerebral glucose metabolism in dementia with Lewy bodies and Alzheimer's disease: a comparative study using positron emission tomography. Neurosci. Lett..

[b0095] Ishii K., Imamura T., Sasaki M., Yamaji S., Sakamoto S., Kitagaki H., Hashimoto M., Hirono N., Shimomura T., Mori E. (1998). Regional cerebral glucose metabolism in dementia with Lewy bodies and Alzheimer's disease. Neurology.

[b0100] Koeppe R.A., Gilman S., Joshi A., Liu S., Little R., Junck L., Heumann M., Frey K.A., Albin R.L. (2005). 11C-DTBZ and 18F-FDG PET measures in differentiating dementias. J. Nucl. Med. Off. Publ. Soc. Nucl. Med..

[b0105] Mosconi L., Tsui W.H., Herholz K., Pupi A., Drzezga A., Lucignani G., Reiman E.M., Holthoff V., Kalbe E., Sorbi S., Diehl-Schmid J., Perneczky R., Clerici F., Caselli R., Beuthien-Baumann B., Kurz A., Minoshima S., de Leon M.J. (2008). Multicenter standardized 18F-FDG PET diagnosis of mild cognitive impairment, Alzheimer's disease, and other dementias. J. Nucl. Med. Off. Publ. Soc. Nucl. Med..

[b0110] Pillai J.A., Wu G., Tousi B., Larvie M., Leger G.C., Leverenz J.B. (2019). Amygdala sign, a FDG-PET signature of dementia with Lewy Bodies. Parkinsonism Relat. Disord..

[b0115] Kantarci K., Lowe V.J., Chen Q., Przybelski S.A., Lesnick T.G., Schwarz C.G., Senjem M.L., Gunter J.L., Jack C.R., Graff-Radford J., Jones D.T., Knopman D.S., Graff-Radford N., Ferman T.J., Parisi J.E., Dickson D.W., Petersen R.C., Boeve B.F., Murray M.E. (2020). beta-Amyloid PET and neuropathology in dementia with Lewy bodies. Neurology.

[b0120] Kantarci K., Lesnick T., Ferman T.J., Przybelski S.A., Boeve B.F., Smith G.E., Kremers W.K., Knopman D.S., Jack C.R., Petersen R.C. (2016). Hippocampal volumes predict risk of dementia with Lewy bodies in mild cognitive impairment. Neurology.

[b0125] Oppedal K., Ferreira D., Cavallin L., Lemstra A.W., Ten Kate M., Padovani A., Rektorova I., Bonanni L., Wahlund L.O., Engedal K., Nobili F., Kramberger M., Taylor J.P., Hort J., Snaedal J., Blanc F., Walker Z., Antonini A., Westman E., Aarsland D. (2019). Alzheimer's Disease Neuroimaging I: A signature pattern of cortical atrophy in dementia with Lewy bodies: A study on 333 patients from the European DLB consortium. Alzheimers Dement..

[b0130] Graff-Radford J., Lesnick T.G., Boeve B.F., Przybelski S.A., Jones D.J., Senjem M.S., Gunter J.G., Ferman T.J., Knopman D.S., Murray M.E., Dickson D.W., Sarro L., Jack C.R., Petersen R.C. (2016). K K: Predicting survival in Dementia with Lewy Bodies with hippocampal volumetry. Movement Disorders Off. J. Movement Disorder Soc..

[b0135] Kantarci K., Ferman T.J., Boeve B.F., Weigand S.D., Przybelski S., Vemuri P., Murray M.E., Senjem M.L., Smith G.E., Knopman D.S., Petersen R.C., Jack C.R., Parisi J.E., Dickson D.W. (2012). Focal atrophy on MRI and neuropathologic classification of dementia with Lewy bodies. Neurology.

[b0140] Villain N., Desgranges B., Viader F., de la Sayette V., Mezenge F., Landeau B., Baron J.C., Eustache F., Chetelat G. (2008). Relationships between hippocampal atrophy, white matter disruption, and gray matter hypometabolism in Alzheimer's disease. J. Neurosci..

[b0145] Kantarci K., Avula R., Senjem M.L., Samikoglu A.R., Zhang B., Weigand S.D., Przybelski S.A., Edmonson H.A., Vemuri P., Knopman D.S., Ferman T.J., Boeve B.F., Petersen R.C., Jack C.R. (2010). Dementia with Lewy bodies and Alzheimer disease: Neurodegenerative patterns characterized by DTI. Neurology.

[b0150] Thomas A.J., Donaghy P., Roberts G., Colloby S.J., Barnett N.A., Petrides G., Lloyd J., Olsen K., Taylor J.P., McKeith I., O'Brien J.T. (2019). Diagnostic accuracy of dopaminergic imaging in prodromal dementia with Lewy bodies. Psychol. Med..

[b0155] Ruppert M.C., Greuel A., Tahmasian M., Schwartz F., Sturmer S., Maier F., Hammes J., Tittgemeyer M., Timmermann L., van Eimeren T., Drzezga A., Eggers C. (2020). Network degeneration in Parkinson's disease: multimodal imaging of nigro-striato-cortical dysfunction. Brain.

[b0160] Petersen R.C., Aisen P., Boeve B.F., Geda Y.E., Ivnik R.J., Knopman D.S., Mielke M., Pankratz V.S., Roberts R., Rocca W.A., Weigand S., Weiner M., Wiste H., Jack C.R. (2013). Mild cognitive impairment due to Alzheimer disease in the community. Ann. Neurol..

[b0165] Kantarci K., Senjem M.L., Lowe V.J., Wiste H.J., Weigand S.D., Kemp B.J., Frank A.R., Shiung M.M., Boeve B.F., Knopman D.S., Petersen R.C., Jack C.R. (2010). Effects of age on the glucose metabolic changes in mild cognitive impairment. AJNR Am. J. Neuroradiol..

[b0170] Roberts R.O., Geda Y.E., Knopman D.S., Cha R.H., Pankratz V.S., Boeve B.F., Tangalos E.G., Ivnik R.J., Rocca W.A., Petersen R.C. (2012). The incidence of MCI differs by subtype and is higher in men: The Mayo Clinic Study of Aging. Neurology.

